# Editorial: Methods and applications in personality and social psychology: The person-environment interaction: New instruments and their first applications

**DOI:** 10.3389/fpsyg.2023.1159841

**Published:** 2023-03-02

**Authors:** Sofya K. Nartova-Bochaver, Tjeerd Andringa

**Affiliations:** ^1^School of Psychology, HSE University, Moscow, Russia; ^2^SoundAppraisal BV, Groningen, Netherlands

**Keywords:** social, natural, urban, built environment, organization, validity, reliability, ecopsychology

People are never free from the influence of the environments in which they are located and never remain inert in relation to them. Blessed are those who have been “thrown” into those environments that fit their personalities. A misfit between a person and the environment makes it difficult to solve current life problems and entails tension and stress, which can even result in mental or physical illness. To assess whether the environment fits a person, it is necessary to have an arsenal of validated research and diagnostic tools. To date, psychology and areas of applied science lack these methods. This collection helps to fill in this gap.

The environment is an integral and indivisible phenomenon. However, due to scientific abstraction, we can distinguish several sub-environments that differ in their origin, purpose, and contribution to human personalities or social relations. So, we can talk about the environment as a physical space, natural and built environments. Nature is primordial; it sets the background of human existence, which determines the finiteness of human life. Nature is not anthropogenic; the enveloping context and precondition encourages us to consider what we cannot control. On the other hand, the connection with nature is a robust psychological resource that makes a person more resilient and empathetic to other living beings.

We can highlight built environments, such as cities, medical and educational organizations, et cetera. People plan these environments, inhabit and personalize them, and express their individuality and resourcefulness. Built environments perform many pragmatic functions. They are easier to change depending on the needs of the people there. Additionally, we can talk about an atmosphere (microclimate) of interaction with the social environment. In this case, the environment will include difficult-to-measure nuances of human relations or the person's attitudes to the world around them.

Not only cities and buildings are man-made, but the digital environment that arose recently has new and pronounced specifics. Having emerged to solve technological and communication problems, the digital environment gave rise to a virtual world that, sometimes, replaces reality for people. Our contemporaries can no longer do without digital technologies but keeping a balance between the real and virtual worlds is important.

Finally, we can talk about the environment as an atmosphere of interaction with the social environment or microclimate. In this case, the environment will include features not always reflected features of a person's attitudes to the world around him.

We are happy to present a series of articles devoted to the development or first use of new methods that reflect different aspects of human interaction with the environment. Larionow et al. focuses on the psychometric properties of the Polish version of the Climate Anxiety Scale and contributed to its general validity.

Another aspect of interaction with nature is presented in the works by Ariccio and Mosca who performed a preliminary adaptation of a very popular Revised Environmental Identity Scale by Clayton in Italy. Environmental identity is a belief that the environment is an important part of who we are. On the sample of the pet-owners, Ariccio and Mosca have discovered that the structure of the scale differs from the initial one and has two factors. This raises a question about the specifics of the environmental identity phenomenon in Italy.

Continuing this investigation line, Rahmani et al. distinguished between environmental identity strength and environmental identity salience. The latter is defined as the frequency of the identity's activation in environmentally relevant everyday life situations. Researchers developed a new instrument, a unifactorial Environmental Identity Salience Scale, consisting of four items. It measures how frequently individuals think of their environmental identity in house-related, transport, waste disposal, and consumption-related activities. In three studies, they have demonstrated that identity strength is less predictive for pro-ecological behaviors compared with identity salience.

Not only natural environments but also built environments need to be investigated and assessed. One of the most influencing the lives of contemporary people is a city with its transport structure. Kochetova investigated drivers' behavior's content, structural, and dynamic aspects by summarizing different observations. She concluded that drivers' traffic behavior patterns in Estonia, Russia, and Kazakhstan differed, demonstrating various styles of person-environment interaction depending on the culture.

Another sample of interaction with the built environment was presented by Kosters et al., who applied the notion of audible safety to show that simply raising awareness in nursing staff on the role of soundscape optimization in long-term care for people with dementia lead to a marked reduction of negatively evaluated soundscapes and an improved care environment. This research goes to the very core of care since care is always high quality when clients feel safe and relaxed.

The environment is not only a physical space; it is also a psychological atmosphere. Bochaver et al. presented a new tool for assessing the school environment, School Climate Questionnaire. The new tool includes 22 items and has three factors: Deviant Behavior, School Well-being, and Subjective Unsafety. It is a valid, reliable, and convenient instrument that seems to be in demand both by researchers and practitioners.

Rózsa et al. measured immersion, involvement, and attention-focusing tendencies in the mediated (digital) environment by studying the applicability of the Immersive Tendencies Questionnaire in association with adaptive and maladaptive personality predispositions.

Finally, whatever environment we consider, general patterns of interaction with it can be described through openness to its information content. The need for cognitive closure determines how people obtain and transmit information and produce judgments, in the first line, from the social environment. Jaume et al. presented an Argentinian adaptation of a reduced version of the Revised Test of Need for Cognitive Closure by Pierro and Kruglanski. They developed a valid, reliable version of the questionnaire consisting of eight items and two factors: Urgency Tendency and Permanence Tendency.

Overall, our RT showed the diversity and importance of the study of Person-Environment Interaction and especially the development of tools to measure and eventually guarantee that the person fits their environment. The topic showed its potential and will develop further since it goes to the very core of being.

## Author contributions

SN-B and TA: wrote the manuscript and approved the submitted version of the manuscript. All authors contributed to the article and approved the submitted version.

